# Freshwater mussels prefer a diet of stramenopiles and fungi over bacteria

**DOI:** 10.1038/s41598-024-62245-2

**Published:** 2024-05-25

**Authors:** Isabella J. Maggard, Kayla B. Deel, Tina W. Etoll, Rachael C. Sproles, Tim W. Lane, A. Bruce Cahoon

**Affiliations:** 1 The University of Virginia’s College at Wise, Wise, VA USA; 2 The Aquatic Wildlife Conservation Center, Virginia Department of Wildlife Resources, Marion, VA USA

**Keywords:** Conservation biology, Food webs, Water microbiology, Animal behaviour

## Abstract

Freshwater mussels (Mollusca: Unionidae) play a crucial role in freshwater river environments where they live in multi-species aggregations and often serve as long-lived benthic ecosystem engineers. Many of these species are imperiled and it is imperative that we understand their basic needs to aid in the reestablishment and maintenance of mussel beds in rivers. In an effort to expand our knowledge of the diet of these organisms, five species of mussel were introduced into enclosed systems in two experiments. In the first, mussels were incubated in water from the Clinch River (Virginia, USA) and in the second, water from a manmade pond at the Commonwealth of Virginia’s Aquatic Wildlife Conservation Center in Marion, VA. Quantitative PCR and eDNA metabarcoding were used to determine which planktonic microbes were present before and after the introduction of mussels into each experimental system. It was found that all five species preferentially consumed microeukaryotes over bacteria. Most microeukaryotic taxa, including Stramenopiles and Chlorophytes were quickly consumed by all five mussel species. We also found that they consumed fungi but not as quickly as the microalgae, and that one species of mussel, *Ortmanniana pectorosa*, consumed bacteria but only after preferred food sources were depleted. Our results provide evidence that siphon feeding Unionid mussels can select preferred microbes from mixed plankton, and mussel species exhibit dietary niche differentiation.

## Introduction

There are approximately 298 native species of freshwater mussels (Bivalvia: Unionida: Unionidae) in the United States and Canada, the majority of which are endangered or threatened, making them one of the most imperiled groups of organisms in North America^1–4^. Population decline is mostly attributed to various anthropogenic impacts such as poor water quality, habitat loss, pollution, and the introduction of invasive species^5–7^. Additionally, enigmatic mass mortality events have been occurring since the late 1960s which cannot be linked to a specific human activity or a microbial pathogen^8–10^. These declines in Unionid populations are particularly concerning because of the important role they play as ecosystem engineers^11^ and in providing significant ecological services that maintain the health of streams and rivers^4,9,12–14^.

Freshwater mussels often live in highly diverse multi-species aggregations which suggests that niche differentiation occurs via selective feeding on different types of microbes to reduce competition. While selective feeding has been documented in marine bivalves such as *Mytilus edulis*^15–18^, freshwater mussel diets are less well understood. Freshwater mussels feed by siphoning suspended plankton from the water column and/or from the sediment. Isotope, biochemical marker, and water clearance rate studies have demonstrated and/or suggested that mussels have a “multi-sourced and opportunistic” diet^19^ consisting of diatoms (Bacillariophyceae), green algae (Chlorophyta), fungal spores, zooplankton, bacteria, and non-living organic matter^19–32^. It is important to understand the particles ingested by freshwater mussels and if there are preferred diet compositions, as this could provide insight into future conservation efforts^33^. For mussel species that are reared in captivity for conservation purposes, food abundance and availability are critical factors, as inadequate diets are clearly harmful to bivalve performance^34^.

This study focused on the dietary preferences of five freshwater mussel species that occur in the Clinch River, Virginia (USA). It is one of the most biodiverse freshwater ecosystems in North America and supports numerous vulnerable mussel populations^7^. Surveys conducted from 2004 to 2009 documented 46 extant freshwater mussel species, including 20 listed as federally endangered of extinction^6^. As understanding the preferred diet composition of freshwater mussels is critical to the success of conservation efforts, we tested two hypotheses, 1—Siphon feeding Unionid mussels can select preferred microbes from mixed plankton, and 2—Different mussel species exhibit dietary niche differentiation.

## Materials and methods

To test these hypotheses, we performed two controlled feeding experiments with mussels raised in captivity at the Virginia Department of Wildlife Resources (DWR), Aquatic Wildlife Conservation Center (AWCC) in Marion, VA. In the first experiment, one of five different species was placed into tanks containing water from the Clinch River and microbial DNA (bacteria, microeukaryotic, and fungal) residing in the water was analyzed before and after a 24 h incubation using quantitative PCR and metabarcoding. In the second experiment, water from a retainment pond at the AWCC was placed into tanks and microbial DNA was analyzed at 0, 24, 48, and 96 h after the introduction of mussels.

### Experiment 1

In September 2022 grab samples were collected using 5-gallon buckets (18.9 L) to scoop water from the Clinch River in St. Paul, VA (USA). Water was poured into 50-gallon (189.2 L) tanks and transported ~ 100 m to UVA Wise’s Oxbow Center. Within 2 h of collection 10 L of river water were placed into eighteen 10-gallon (37.8 L) fish tanks with no substrate. Water temperature, pH, and conductivity was measured using Orion portable meters (ThermoFisher, Waltham, MA). Three one-liter samples of Clinch River water were passed through 0.45-μm filters (Cytiva, Marlborough, MA, USA) to collect microbes for DNA extraction and serve as time zero samples. Filters were immediately removed from the funnel and placed into a bead-beater tube containing the extraction buffer supplied in a PowerWater DNA extraction kit (Qiagen, Germantown, MD, USA), shaken to distribute the buffer onto the filter and stored at − 20 °C for 8 days. An equal number of one species of mussel was introduced into three replicate tanks (Table 1). Five total species were tested, *Ortmanniana pectorosa *(previously *Actinonaias pectorosa*), *Ortmanniana ligamentina *(previously *Actinonaias ligamentina*), *Lampsilis fasciola, Lampsilis ovata,* and *Cambarunio iris*. The mussels used in this experiment were bred in captivity at the AWCC where they were sustained on a live planktonic diet received as a continuous flow from pond water sieved below 300 μm. All mussels were left to purge pseudofeces for 24 h prior to the start of the experiments. The mussels were transported to St. Paul, VA the day of the experiment in aerated coolers filled with culture water filtered to 5 μm. The species used in this study were chosen because they naturally occur in the Clinch River, were abundantly available at the AWCC, and are not listed as endangered or threated. Prior to introduction, forty-five individuals of each species (15 from each replicate tank) were measured with digital calipers (Table 1). All tanks were mechanically aerated through 6 mm tubing connected to air stone cylinders. Three control tanks contained no mussels. All tanks were incubated 24-h in a room with the ambient temperature set to match the river’s water temperature at the time of removal, 21.1 °C (70 °F). After the 24-h incubation, the water in each tank was thoroughly mixed to resuspend debris and pseudo-feces that had collected onto the bottoms of the tanks. Water temperature, pH, and conductivity was measured and one-liter samples of water were removed from each tank and passed through a filter funnel with a 0.45-μm membrane. Filters were immediately removed from the funnel and placed into a bead-beater tube containing extraction buffer, shaken, and stored at − 20 °C for 7 days. All frozen filters coated in extraction buffer in the bead beater tubes were thawed in a 65 °C incubator and DNA extractions completed following the manufacturer’s instructions. DNA concentrations were measured using a Nanodrop Lite (ThermoFisher, Waltham, MA).

**Table 1:** The average sizes of each mussel species and the number of individuals placed into each tank.

Species	Mussels per tank	Average shell lengths (mm) ± SD		
		Tank 1	Tank 2	Tank 3
Experiment 1—Clinch River water—mussel sizes				
*Ortmanniana ligamentina*	60	15.96 ± 0.88	15.82 ± 1.35	15.0 ± 0.91
*Ortmanniana pectorosa*	60	16.3 ± 1.06	16.03 ± 1.35	16.26 ± 1.41
*Lampsilis fasciola*	45	16.1 ± 1.71	15.5 ± 0.85	15.73 ± 0.98
*Lampsilis ovata*	30	16.13 ± 1.29	16.26 ± 1.07	15.9 ± 2.56
*Cambarunio iris*	60	16.4 ± 1.45	15.77 ± 1.01	15.28 ± 2.54
Control/no mussels	0	0	0	0
Experiment 2—AWCC retaining pond water—mussel sizes				
*Ortmanniana ligamentina*	20	19.8 ± 1.18	19.36 ± 1.00	18.29 ± 1.93
*Ortmanniana pectorosa*	20	19.9 ± 3.45	21.01 ± 1.73	20.45 ± 3.44
*Lampsilis fasciola*	20	17.93 ± 3.24	18.4 ± 1.84	18.89 ± 1.82
*Lampsilis ovata*	20	21.69 ± 4.62	19.96 ± 3.98	21.9 ± 4.85
*Cambarunio iris*	20	17.4 ± 3.40	18.94 ± 2.45	18.39 ± 3.92
Control/no mussels	0	0	0	0

Statistical comparisons of conductivity and pH between control tanks and individual species were conducted with one-way ANOVA and Kruskall–Wallis tests with multiple comparisons using GraphPad Prism v10.1.0 (GraphPad Software, Boston, MA, USA). A summary of replicates, statistical tests, and p-values can be found in Supplementary Table S1. A significant difference was defined as p ≤ 0.05.

#### Quantitative PCR

Quantitative PCR (qPCR) analysis was performed using a Bio-Rad CFX96 C1000 Thermal Cycler and SsoAdvanced SYBR Green Supermix (BioRad, Hercules, CA, USA). Prokaryotic 16S V4 regions were detected using ‘universal’ prokaryotic primers, 764F (5′CAAACAGGATTAGATACCC) and 1061R (5′CCGTCAATTCCTTTRAGTTT) which produces an amplicon ~ 150 bp^35^. The eukaryotic 18S V4 region was detected using ‘universal’ microeukaryote primers, TAReuk454FWD1 (5′CCAGCASCYGCGGTAATTCC) and TAReukREV3 (5′ACTTTCGTTCTTGATYRA) which produces an amplicon ~ 425 bp^36^. Fungal rRNA ITS2 region was detected using (ITS86_F 5′-GTGAATCATCGAATCTTTGAA, ITS4_R 5′-TCCTCCGCTTATTGATATGC) which produces an amplicon ~ 275 bp^37–39^. DNA collected from each tank (representing 3 independent experimental replicates per species, n = 3) was each assessed with 3 replicate reactions (technical replicates) for a total of 9 readings for each mussel species. Crossover threshold values were converted to DNA copy number using methodology described in Grimes et al.^40^. Technical replicates from each independent replicate were averaged and then the three independent replicates were averaged. Statistical comparisons between the control tanks and individual species were conducted with one-way ANOVA and Kruskall–Wallis tests with multiple comparisons using GraphPad Prism v10.1.0. A summary of replicates, statistical tests, and p-values can be found in Supplementary Table S1.

#### Metabarcoding

Microeukaryotic, fungal and prokaryotic barcodes were PCR amplified using the primers described above for qPCR. Amplicons were produced from each sample in triplicate using Phusion DNA polymerase (ThermoFisher, Waltham, MA, USA) with the provided High Fidelity buffer, 5 pmol of each primer, and a BioRad C1000 thermal cycler programmed to heat to 95 °C 10 mins, (95 °C 30 s, 55 °C 30 s, 72 °C 30 s) × 40 cycles, followed by a 72 °C 10 min soak. Amplicon production was confirmed by gel electrophoresis. Triplicate technical replicates were combined to form a single mixture. Amplicons from each sample were prepared for sequencing using the GeneJET PCR Purification kit and sequenced by Azenta (South Plainfield, NJ, USA) using their Amplicon-EZ service. Illumina paired-end raw sequence data were assembled and sequence quality control performed with DADA2 (truncLen = c(250,250) for protist 18S and fungal ITS and truncLen = c(125,125) for 16S) in the QIIME2 environment, v. 2022.8^41^. For beta-analysis non-metric multidimensional scaling (NMDS) based on Bray-Curtis dissimilarity distances was completed using metabarcoding data from three independent replicates (i.e. three separate tanks for each experiment) for species + control for each set of markers—16S, 18S, or fungal ITS) using QIIME2. NMDS plots were generated with EMPeror^42^. Differences in community structure were tested using permutational analysis of variance tests (PERMANOVA) with 999 permutations in QIIME2.

Raw data were also paired, Amplicon Sequence Variants (ASVs) generated and taxonomically identified using DADA2^43^ in R with default parameters and truncLen = c(250,250) for protist 18S and fungal ITS and truncLen = c(125,125) for 16S. Taxonomic predictions were made using the Silva, v.138.1, UNITE ITS, v.29.11.2022, and pr2, v.4.14 databases for 16S, ITS, and 18S, respectively^44–49^.

### Experiment 2

A second controlled feeding experiment was conducted in May 2023 at the AWCC. Water was pumped directly from an outdoor ½ acre (2023 m^2^) pond into eighteen 10-gallon (37.8 L) fish tanks. This manmade pond is maintained and managed by AWCC staff to provide live microbial feed for mussels. 20 L of pond water (double the amount used in the first experiment) were placed into each tank and mechanically aerated through 6 mm tubing connected to air stone cylinders. The same five mussel species were introduced into tanks and three control tanks received no mussels as described for experiment 1. The numbers of mussels introduced into each tank differed from the first experiment, 20 to each tank (Table 1). The average sizes are listed in Table 1. Tanks with mussels were incubated in an enclosed building with no heating or cooling so the mussels were subject to ambient temperatures. Tank temperatures, pH, and conductivity were measured as described above. Average water temperatures are listed in Supplementary Table S2.

For DNA analysis, 3, 1 L samples of water pumped from the retention pond were filtered at the onset of the experiment to represent Time 0. One liter samples of water were removed from all tanks after 24, 48, and 96 h of incubation. Before removal, tank water was thoroughly mixed to resuspend sediment and pseudo-feces. Water samples were passed through 0.45 µm filters which were immediately placed into DNA extraction buffer as described above. DNA extractions, PCR, sequencing, qPCR, and bioinformatics were conducted as described above for Experiment 1. The planktonic microbiome of the retention pond was also sampled at the beginning of the experiment using a 20 μm plankton net and examined microscopically.

The same statistical tests were used as described for Experiment 1 and a summary of replicates, statistical tests, and p-values can be found in Supplementary Table S3.

#### Ethics declaration

All mussels used in this study were bred in captivity at the AWCC and required no permitting for their use. No mussels were harmed or sacrificed during the feeding studies. The feeding experiments were conducted using established protocols for culturing these species. Guidelines outlined in the ARRIVE protocol for the use of live animals (https://arriveguidelines.org) were followed in the preparation of this manuscript.

## Results

### Experiment 1—Clinch River water

#### Mussel effects on water chemistry

**Figure 1:**
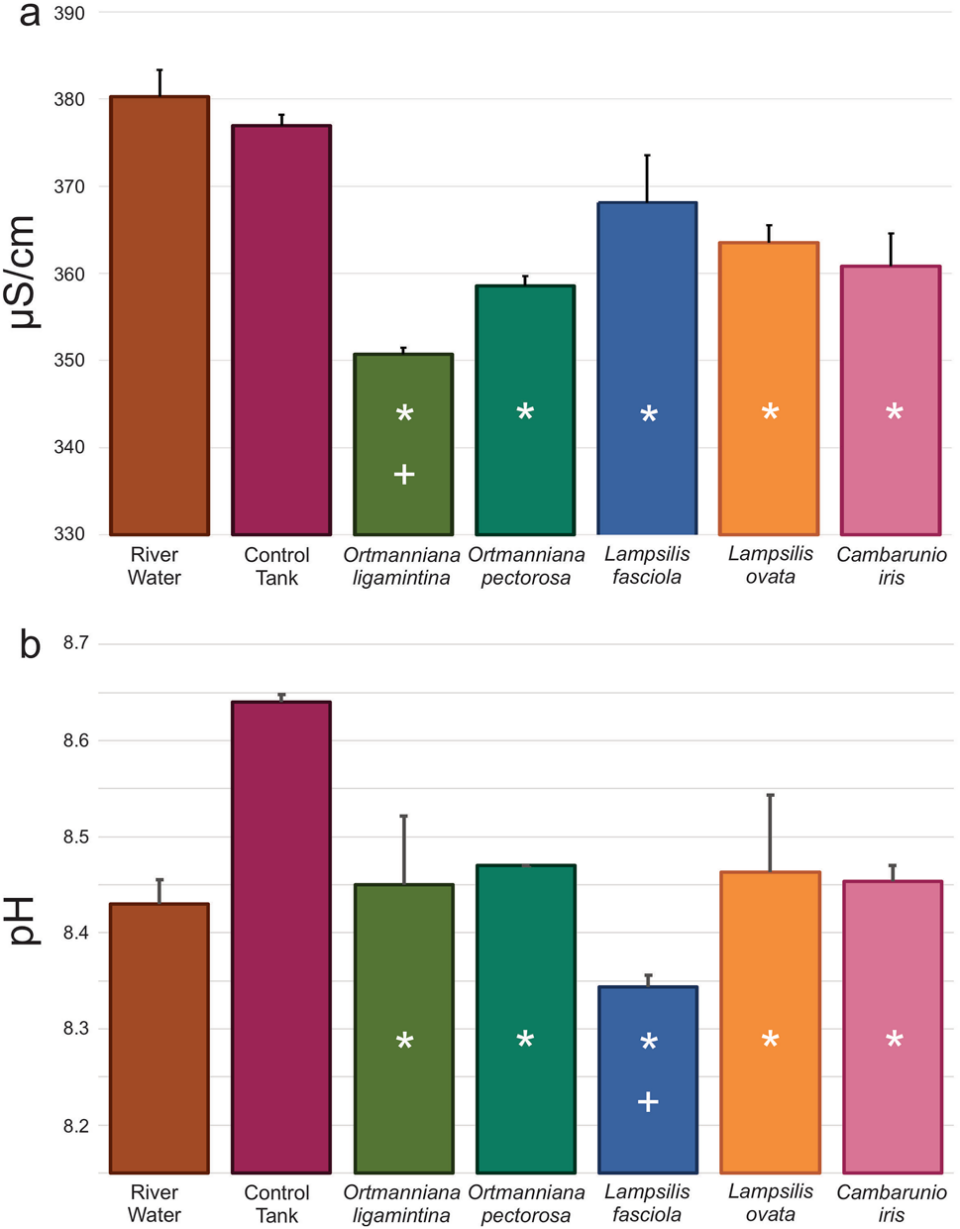
Conductivity and pH of Clinch River Water Before and After Incubation with Mussels. River water conductivity (**a**) and pH (**b**) was measured immediately after being placed into tanks and 24 h after the introduction of mussels. Control tanks contained no mussels. Error bars represent standard deviation. Asterisks represent p-values equal or lower than 0.05 compared to river water (one-way ANOVA). Plus symbols represent p-values equal or lower than 0.05 compared to river water (one-way Kruskal–Wallis).

Water conductivity was statistically lower in tanks containing mussels after the 24-h period when compared to either initial river water samples or tanks containing no mussels (Fig. 1a, Supplementary Table S1a). Tanks containing the species *O. ligamentina* had the most significant decrease in conductivity. Water pH significantly increased in the control tanks with no mussels compared to the river water collected 24 h earlier. All tanks with mussels had a pH that was significantly lower than the no-mussel control tanks but only tanks containing *L. fasciola* were significantly lower than the river water (Fig. 1a, Supplementary Table S1b).

#### qPCR measurements of the Clinch River microbiome

**Figure 2:**
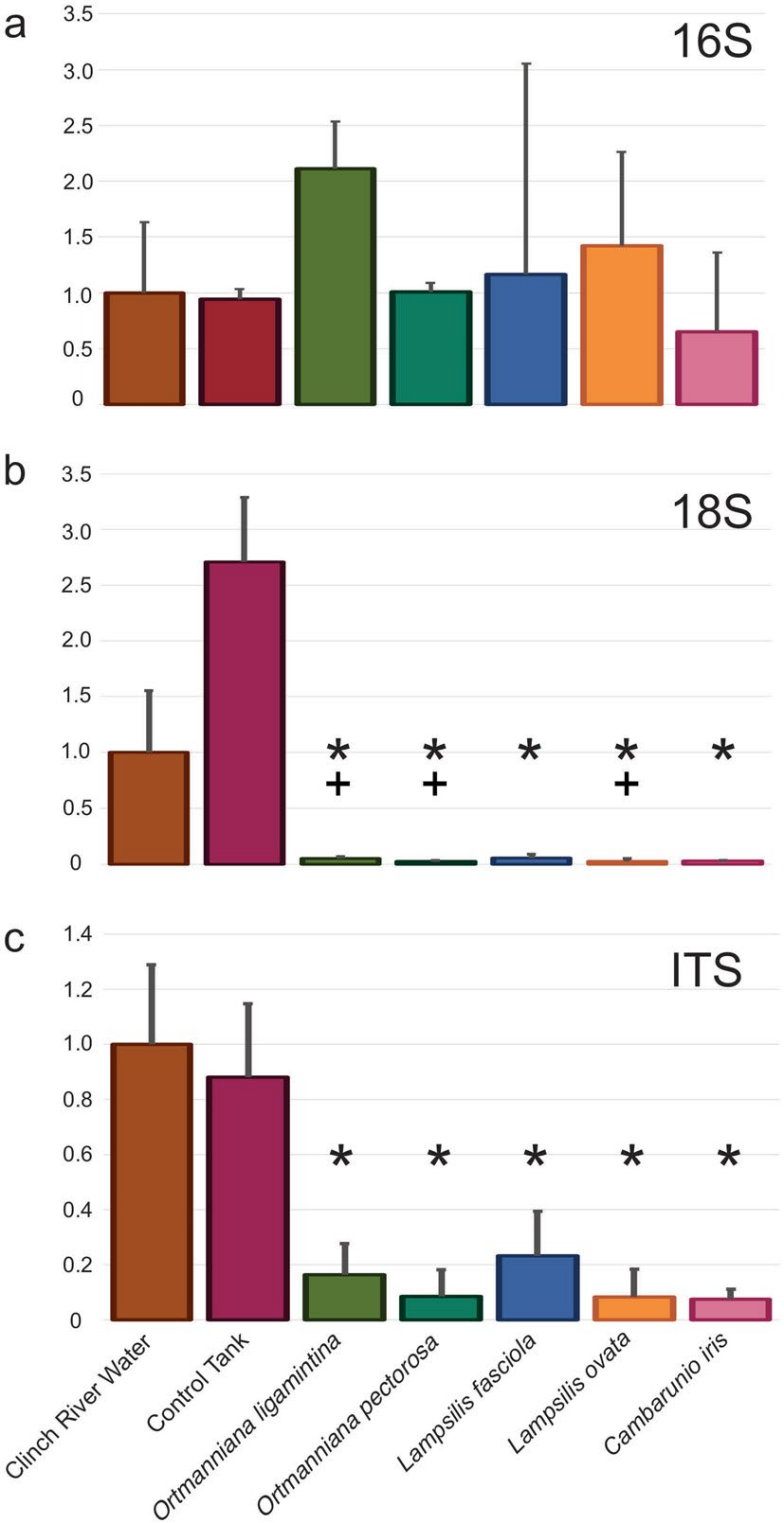
Estimation of the Clinch River planktonic microbiome using quantitative PCR. (**a**) Bacterial 16S. (**b**) Microeukaryotic 18S. (**c**) Fungal ITS. For each experiment, the amount of microbial DNA detected in each sample is expressed as a value relative to river water which was arbitrarily set to a value of 1.0. River water represents three water samples collected from the Clinch River at the beginning of the experiment. Three control tanks received no mussels and water samples were collected 24 h after its introduction. One species of mussel was placed into three replicate tanks with river water and incubated for 24 h before water samples were removed. Error bars represent standard deviation. Asterisks represent p-values equal or lower than 0.05 compared to river water (one-way ANOVA). Plus symbols represent p-values equal or lower than 0.05 compared to river water (one-way Kruskal–Wallis).

Quantitative PCR was used to broadly measure the presence of bacteria (16S), microeukaryotes (18S) and fungi (ITS) in river water before and after incubation with the five mussel species. The amount of available 16S DNA did not significantly change when mussels were present (Fig. 2a, Supplementary Table S1c). Both 18S (Fig. 2b, Supplementary Table S1d) and ITS (Fig. 2c, Supplementary Table S1e) signals significantly decreased after a 24 h incubation with any of the mussel species.

#### Metabarcoding assay of the Clinch River microbiome

**Figure 3:**
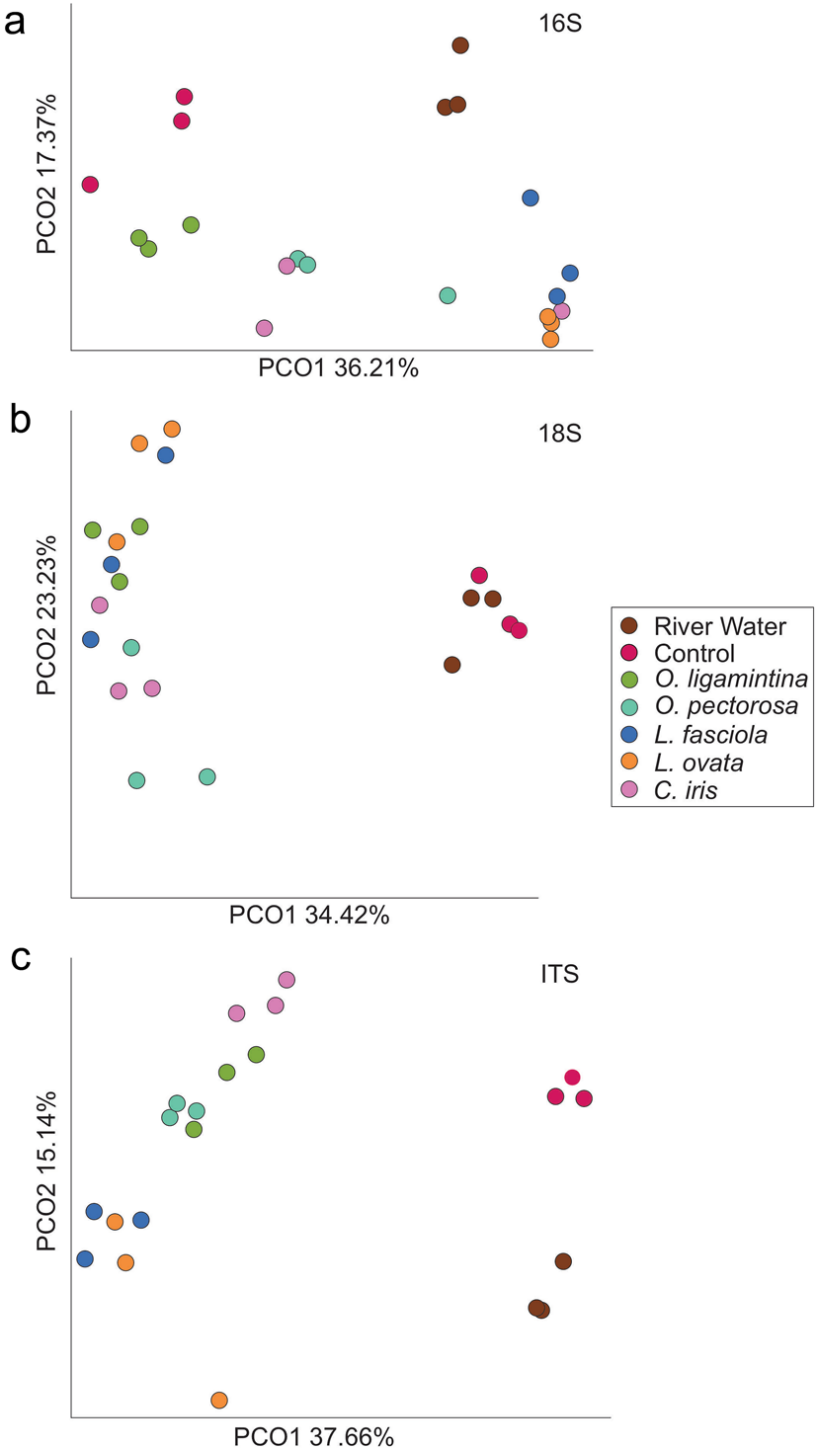
Beta diversity comparisons of the bacterial, microeukaryotic, and fungal portions of the Clinch River planktonic microbiome as principle coordinates plots. (**a**) 16S bacterial barcodes. (**b**) 18S microeukaryotic barcodes. (**c**) Fungal ITS directed barcode markers. River Water represents the microbiome of Clinch River water collected at the beginning of the experiment. Control represents river water incubated in three tanks for 24 h with no mussels. Each species name represents the mussel that was introduced into three replicate tanks and incubated for 24 h before water samples were analyzed. Species are color-coded.

Prokaryotic, microeukaryotic, and fungal metabarcoding beta diversity analyses demonstrate overall significant differences between controls (river water and tanks with no mussels) and tanks with mussels (PERMANOVA pseudo-F test statistics: prokaryotic = 5.41, microeukaryotic = 5.62, fungal = 10.487, all with p < 0.001). PCoA plots revealed a separation between the mussel species along axis1. A separation between tanks with and without mussels on axis 2 demonstrate there were some mussel specific changes to the prokaryotic microbiome (Fig. 3a). Microeukaryotic and fungal beta diversity PCoA plots demonstrated a clear separation between control versus mussel containing tanks along axis 1 and some mussel species specific differences along axis 2 (Fig. 3b,c)

#### Composition of the Clinch River microbiome and mussel dietary preferences

**Figure 4:**
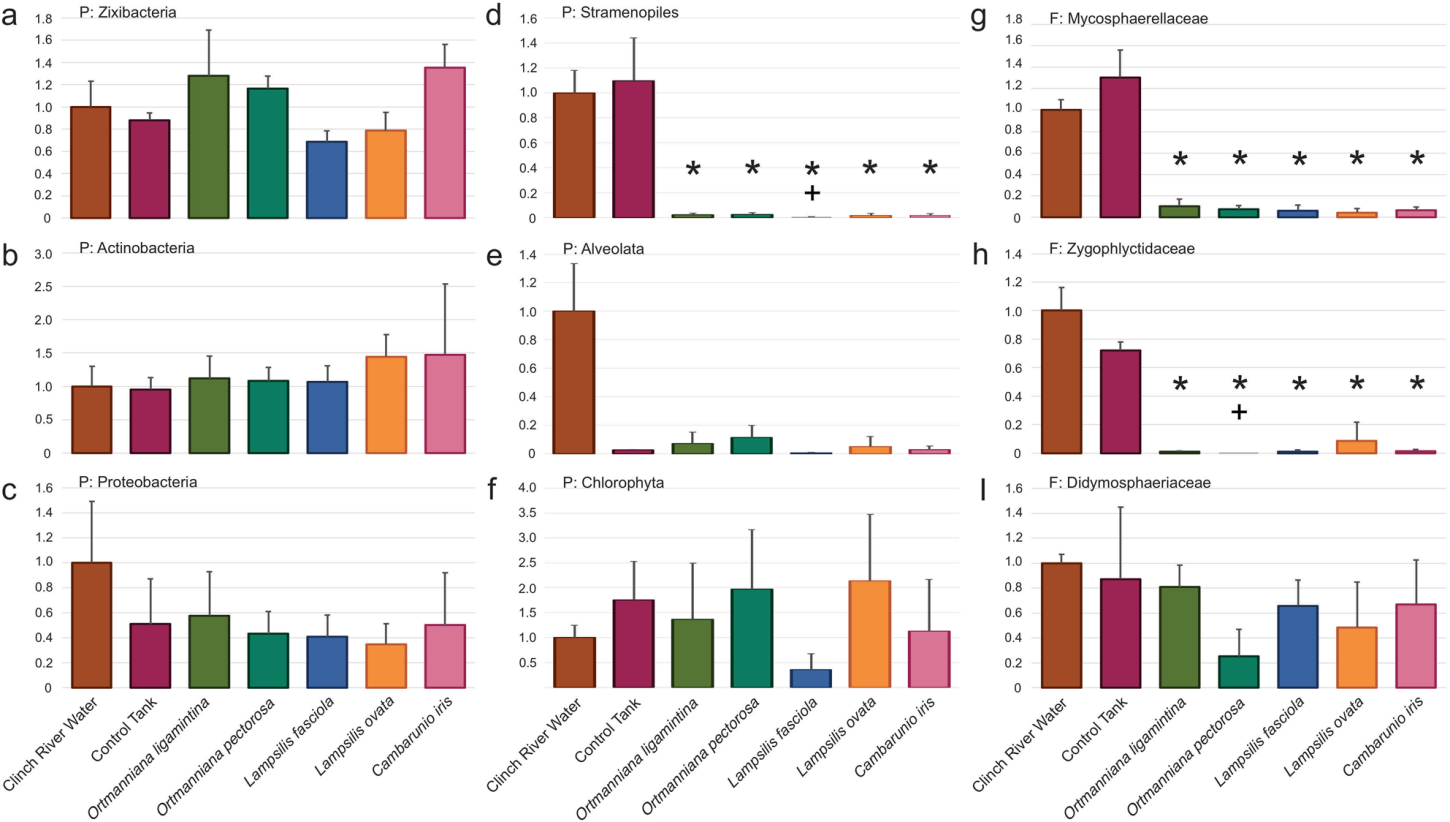
Taxonomic composition of the planktonic microbiome in the Clinch River before and after incubation with mussels. (**a**–**c**) Bacterial phyla. (**d**–**f**) Microeukaryotic phyla. (**g**–**i**) Fungal families. Metabarcode sequences were used to identify taxa present in water samples. Metabarcode read counts were used to estimate the relative amounts of each taxon the mussels had cleared from the plankton within a 24 h incubation. The proportion of reads attributed to each taxon are expressed as values relative to those found in the Clinch River at the initiation of the experiment, which is arbitrarily set to the value of 1.0.

Sixteen prokaryotic phyla were detected among the metabarcoding reads (Supplementary Table S4) with over 99% belonging to three phyla, Zixibacteria = 67.3%, Actinobacteria = 25.1%, and Proteobacteria = 6.8%. There were no significant differences between the relative amounts of the dominant phyla found in water collected from the Clinch River, the control tanks with no mussels, and the tanks containing one of the mussel species (Fig. 4a–c, Tables S1f–h). Analyses of lower taxonomic groups also revealed insignificant differences.

Seven broad microeukaryotic taxonomic groups were detected but over 94% of the total reads belonged to three groups—Stramenopiles = 82.7%, Alveolata = 8.7% and Chlorophyta = 2.8% (Supplementary Table S5). Stramenopiles were abundant in river water samples and remained high in control tanks, but were barely detectable 24 h after mussels were introduced into tanks (Fig. 4d, Supplementary Table S1i). The most abundant of the Clinch River planktonic stramenopiles was Synurales, followed by Pseudodendromonadales, and Bacillariophytes. All three groups were significantly reduced by the presence of mussels. Alveolates were present in river water and clearly missing in tanks with mussels but they were also missing in the Control Tanks so it is unclear if the mussels consumed the Alveolates or if they succumbed to the experimental conditions (Fig. 4e, Supplementary Table S1j). Chlorophyta were present in river water, Control Tanks, and in tanks containing four of the five mussel species. The exception was tanks containing the mussel species *L. fasciola* where the amount of Chlorophyta reads were significantly reduced (Fig. 4f, Supplementary Table S1k).

Seven fungal phyla were detected but at that taxonomic level there were no clear differences in the ITS reads in the river water, control tanks, or those with mussels. At the family level, however, distinct patterns could be seen (Supplementary Table S6). Four hundred and six fungal taxonomic families were detected from water collected from the Clinch River with 79.3% of the total number of reads belonging to three—Mycosphaerellaceae = 46.2%, Zygophlyctidaceae = 19.1%, and Didymosphaeriaceae = 14.0.% (Supplementary Table S6). Species within the Mycosphaerellaceae and Zygophlyctidaceae families comprised the majority of the fungi in river water and were present at comparable levels in Control Tank samples but were significantly lower if mussels were present (Fig. 4g,h, Tables S1l,m). Species within Didymosphaeriaceae were present at comparably high levels in river water, Control tanks, and four of the mussel samples, but were depleted in tanks with *O. pectorosa* (Fig. 4I, Supplementary Table S1n). Species within two other families, Didymellaceae and Cladosporiaceae, comprised ~ 3% and ~ 2% of the fungi in the Clinch River Water, respectively, but the introduction of mussels did not significantly affect their levels of detectable DNA (Supplementary Fig. S2).

### Experiment 2—AWCC

#### Mussel effects on water chemistry

**Figure 5:**
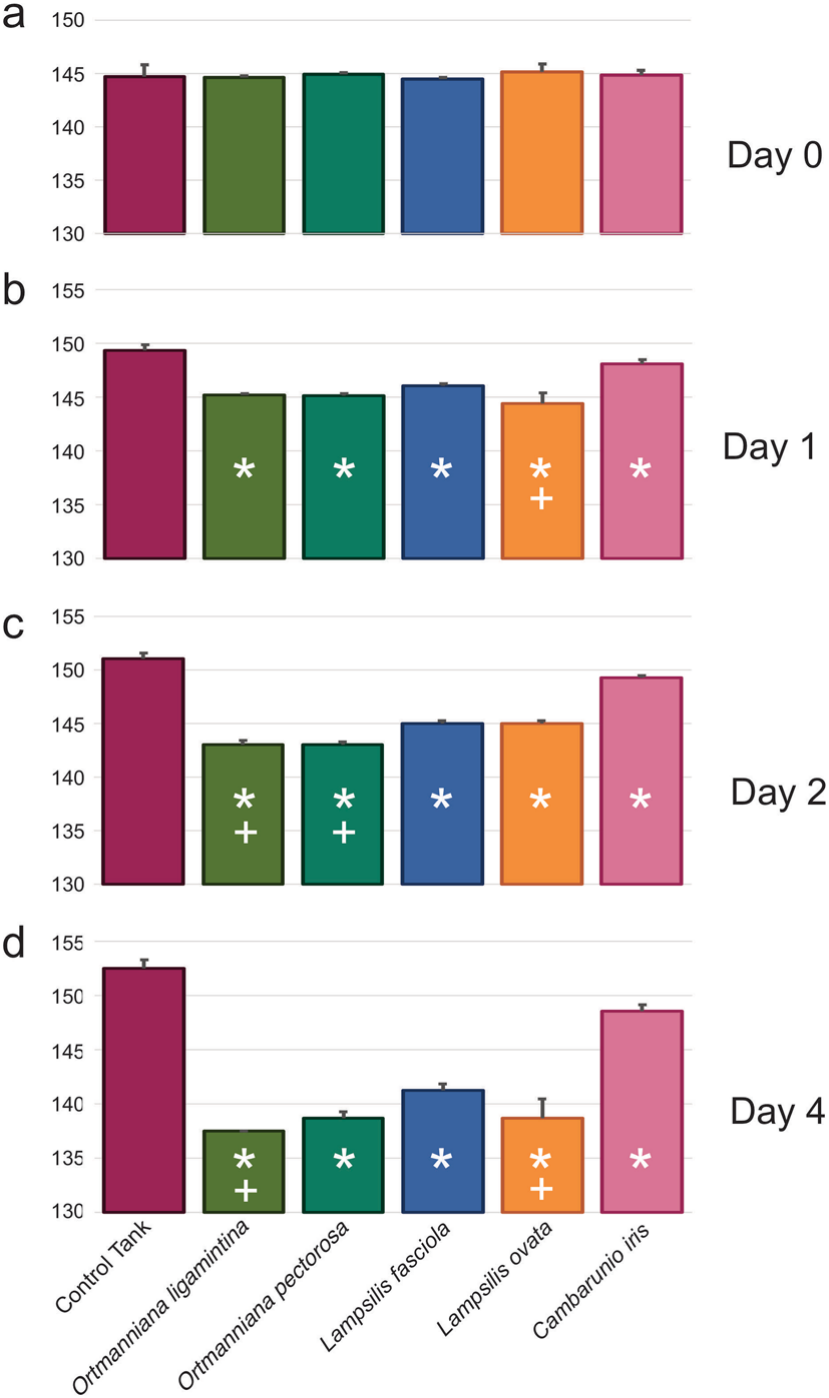
Conductivity of the AWCC retention pond water before and after incubation with mussels. (**a**) Conductivity was measured immediately after the retention pond water was placed into each tank used in this experiment. Control tanks received no mussels. (**b**) Conductivity as measured 24 h after mussels were introduced. (**c**) Conductivity as measured 48 h after mussels were introduced. (**d**) Conductivity as measured 96 h after mussels were introduced. Error bars represent standard deviation. Asterisks represent p-values equal or lower than 0.05 compared to river water (one-way ANOVA). Plus symbols represent p-values equal or lower than 0.05 compared to river water (one-way Kruskal–Wallis).

When mussels were incubated in water from a retention pond at the AWCC all five species significantly reduced the conductivity of the water after a 24 h incubation (Fig. 5a,b, Tables S3a,b) as compared to the control tank. This reduction was more pronounced after 48 h (Fig. 5c, Supplementary Table S3c) and 96 h (Fig. 5d, Supplementary Table S3d). Changes in water pH after 96 h after incubation with mussels were inconsequential and can be found in Supplementary Fig. S2.

#### qPCR measurements of the microbiome

**Figure 6:**
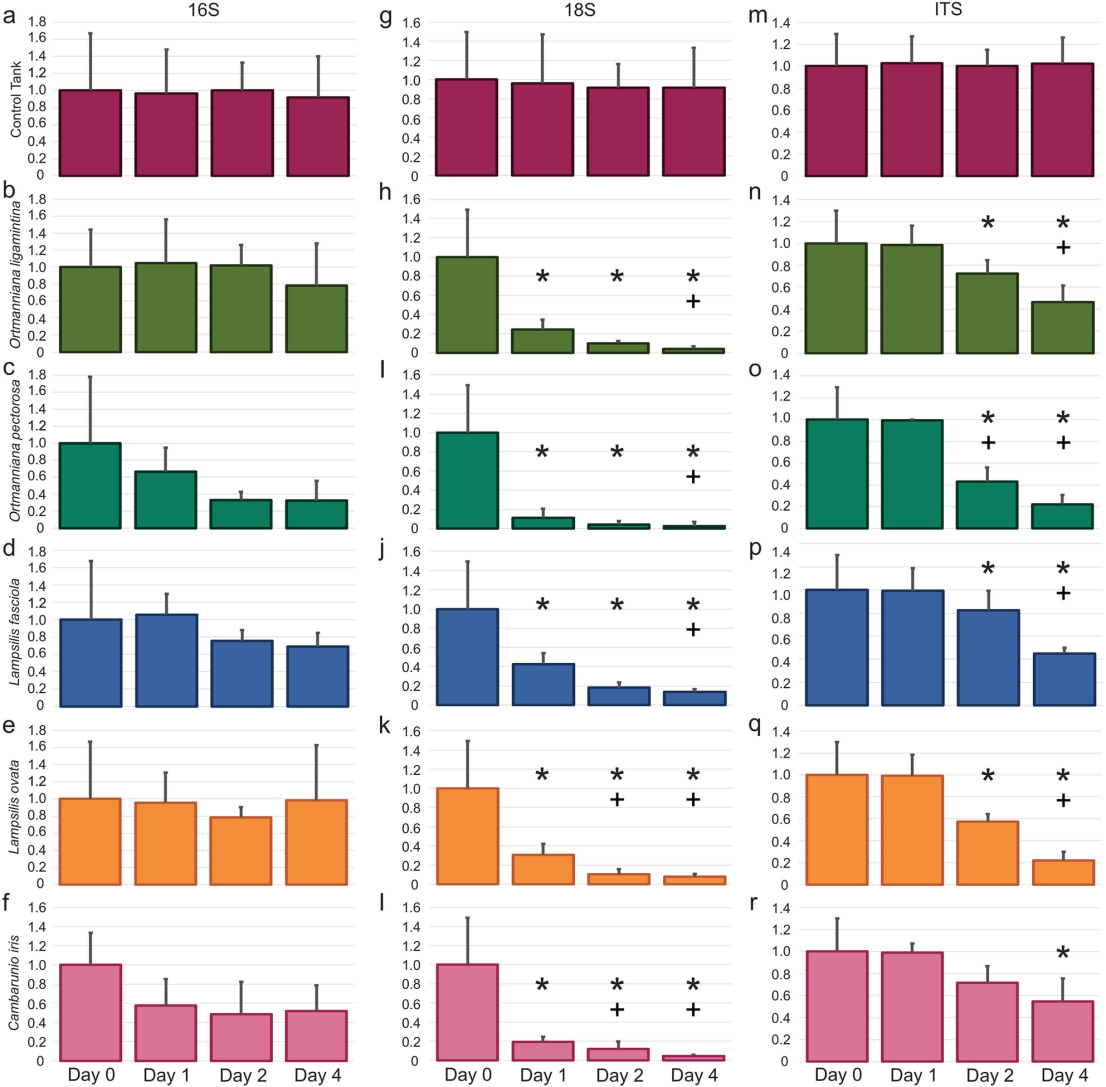
Quantitative PCR estimation of the microbes cleared from the AWCC retention pond plankton over the course of 4 days. (**a**–**f**) Bacterial 16S. (**g**–**l**) Microeukaryotic 18S. (**m**–**r**) Fungal ITS. Water was pumped from the AWCC retention pond into tanks and water samples removed and analyzed (Day 0). Mussel species were placed into the tanks and water samples removed and analyzed one, two, and four days after the beginning of the experiment. Control tanks received no mussels. Error bars represent standard deviation. Asterisks represent p-values equal or lower than 0.05 compared to river water (one-way ANOVA). Plus symbols represent p-values equal or lower than 0.05 compared to river water (one-way Kruskal–Wallis).

There were no significant differences in the amount of 16S bacterial DNA in the tanks with mussels after 24 h of incubation (Fig. 6a–f, Tables S3e–j). For four of the five species this was still the case after 96 h of incubation. Tanks with *O. pectorosa*, on the other hand, did have lower amounts of bacterial DNA in the tanks after 48 and 96 h that were nearly significant, p = 0.07 with an ANOVA test and p = 0.06 with a Kruskall–Wallis test (Fig. 6c, Supplementary Table S3g). All tanks with mussels showed a significant depletion of 18S microeukaryotic DNA within 24 h (Fig. 6g–l, Tables S3k–p). The amount of 18S DNA continued to decrease throughout the 96 h incubation. Fungal ITS DNA decreased in the presence of all five mussel species but there were differences in the rates of depletion. Tanks with *O. ligamentina*, *O. pectorosa, L. fasciola,* and *L. ovata* had significantly lower amounts of ITS DNA within 48 h. Tanks with *C. iris*, on the other hand, were not significantly lower than the control tanks until 96 h after the introduction of the mussels (Fig. 6m–r, Tables S3q–v).

#### Composition of the pond microbiome and mussel dietary preferences

**Figure 7:**
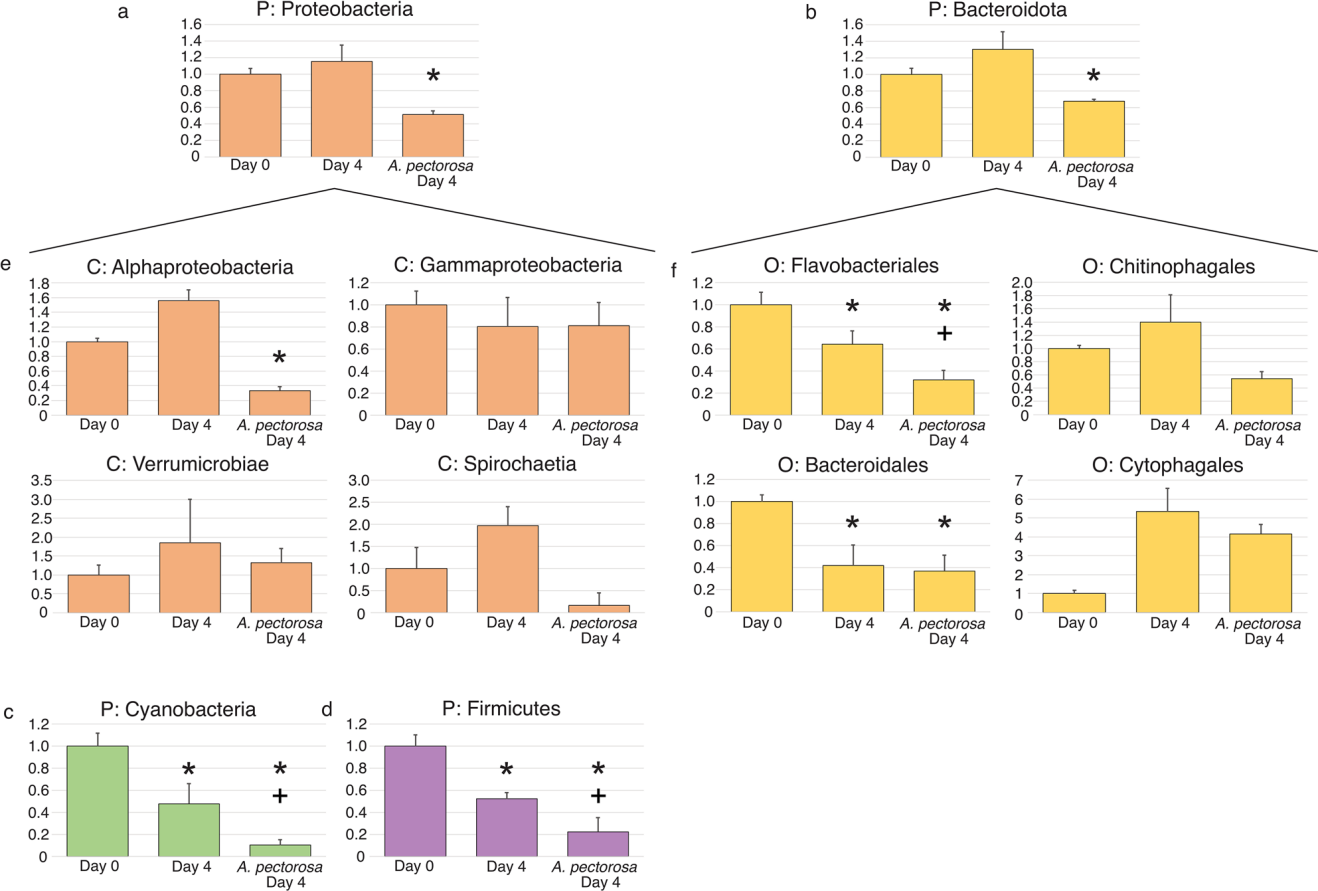
Taxonomic composition of the planktonic bacteria in the AWCC retention pond before and after incubation with *O. pectorosa*. (**a**–**d**) Bacterial phyla. (**e**) The 4 most common classes within the phylum Proteobacteria. (**f**) The 4 most common orders within the phylum Bacteroidota. Metabarcode sequences were used to identify taxa present in water samples when the experiment was set up (Day 0), in a control tank with no mussels (Control, Day 4), and four days after *Ortmanniana pectorosa* was added. The proportion of reads attributed to each taxon are expressed as values relative to those found in the Clinch River at Day 0 of the experiment, which is arbitrarily set to the value of 1.0.

Metabarcoding analysis of pond water revealed that 97.5% of the bacterial microbiome belonged to 4 phyla—Bacteroidota, Proteobacteria, Actinobacteriota, and Cyanobacteria (Supplementary Table S7). Quantitative PCR demonstrated that *O. pectorosa* was the only mussel that cleared bacteria from the plankton so metabarcoding was performed at 0 h and 96 h after incubation with *O. pectorosa*. All four phyla were significantly reduced (Fig. 7a–d, Supplementary Table S3w–z). Analysis of the Proteobacteria phylum at the class level showed that Alphaproteobacteria and Spirochaetia were cleared but not Gammaproteobacteria or Verrucomicrobiae (Fig. 7e, Tables S3aa–ad). Analysis of Bacteroidota at the order level revealed that Flavobacteriales, Bacteroidales, and Chitinophagales were partially cleared but Cytophagales was not (Fig. 7f, Tables S3af–ah).

Metabarcoding and microscopic analyses determined that, at the time of this experiment, the retention pond primarily contained two microalgal species—a diatom (*Fragilaria* sp., 63.4% based on barcode reads) and a green alga (*Desmodesmus* sp., 17.5% based on barcode reads). Due to this clear lack of diversity within the pond, 18S metabarcoding of plankton after incubation with mussels was not performed.

**Figure 8:**
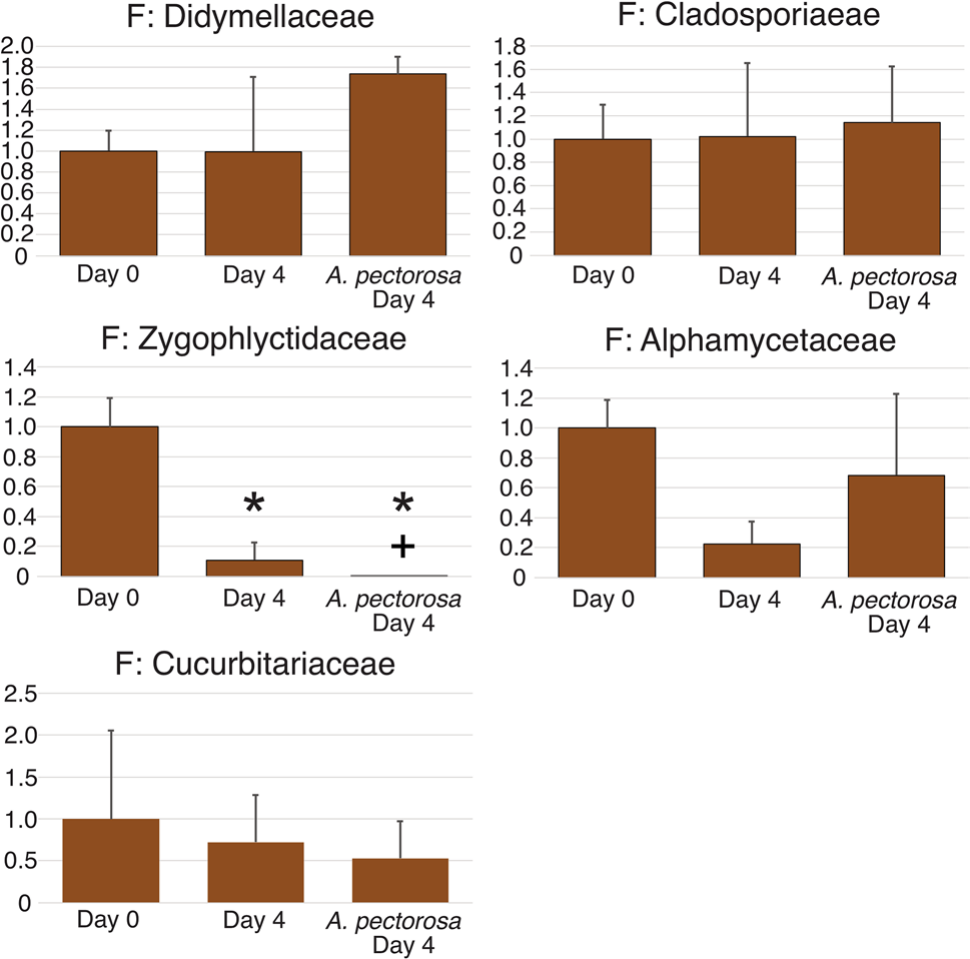
Taxonomic composition of the planktonic fungi in the AWCC retention pond before and after the addition of *O. pectorosa*. The 5 most common fungal families are shown. Metabarcode sequences were used to identify which taxa were present in water samples when the experiment was set up (Day 0), in a control tank with no mussels (Control, Day 4), and four days after *Ortmanniana pectorosa* was added. The proportion of reads attributed to each taxon are expressed as values relative to those found in the Clinch River at Day 0 of the experiment, which is arbitrarily set to the value of 1.0.

Fungi comprised 14.4% of the total microeukaryotic barcode reads (combined 18S and ITS). Within the fungal portion of the microeukaryotic microbiome the majority were from the family Didymosphaeriaceae (~ 68%) with the next most common families being Cladosporiaceae (~ 6.7%), Zygophlyctidaceae (~ 6.5%), Alphamycetaceae (~ 4.5%), and Cucurbitariaceae (~ 2.5%) (Supplementary Table S8). Metabarcoding analysis of ITS metabarcodes at 0 and 96 h after the introduction of *O. pectorosa* only found a significant decline in Zygophlyctidaceae but it also severely declined in the control tanks (Fig. 8, Tables S3ai–am).

## Discussion

We used quantitative PCR and metabarcoding to analyze the planktonic microbiome of water taken from the Clinch River and a pond at the AWCC before and after incubation with Unionid mussels in an enclosed experimental system. Comparisons of microbial constituents in two independent experiments suggest that the mussels exhibited strong selective feeding with a progression of food sources based on availability. Mussels fed first on microalgae with an affinity for Stramenopiles. They also fed on fungi, presumably spores, once microalgae were removed, and one of the species, *O. pectorosa*, fed on bacteria after microalgae and fungi were depleted.

### Algal consumption

Our data demonstrates that freshwater mussels consume both Stramenopiles and Chlorophytes but may exhibit a preference for the former. Freshwater mussels quickly fed upon all Stramenopiles present in the plankton. The mussels cleared *Synura* (Ochrophyta) which was the most abundant Stramenopile present in the Clinch River plankton during experiment 1. They also cleared diatoms (Bacillariophyta) which were present in the plankton in experiments 1 and 2 and was clearly the most abundant microeukaryote in the second experiment as a species of *Fragilaria* was blooming in the pond. The clearing of green algae (Chlorophyta) from river water was less apparent as it was a minor component in the Clinch River the day the water was collected, which made before and after comparisons by metabarcoding less reliable. In experiment 2, however, a considerable proportion of the algae present was the green alga *Desmodesmus* which were eventually cleared/consumed over the course of 4 days. We did not test if the *Desmodesmus* was consumed along with *Fragilaria* or only after *Fragilaria* was depleted.

Several isotope-based studies have shown that freshwater mussels consume microalgae^19,21,30,50^ and it is common for mussels bred in captivity to be fed a mixture of green algae and diatoms^34,51^. A preference for Stramenopiles has been suggested by past experiments. For example, in controlled feeding experiments, *C. iris* juveniles fed a diet which included diatoms (*Nitzschia*, *Cyclotella*, and *Phaeodactylum*) had more robust growth than those exclusively fed green algae (*Chlorella*, *Ankistrodesmus*, and *Chlamydomonas*)^51^. This is presumably due to these diatoms’ ability to accumulate intracellular oil droplets rich in unsaturated fatty acids, a trait attributed to the entire phylum^52^. Techniques for growing some Stramenopile species at large scale have been developed as their propensity for oil accumulation make them attractive for biofuels production^53^. This could benefit mussel propagation as these techniques could be adopted for the on-site production of mussel feed at breeding facilities.

### Fungal consumption

A broad eDNA survey of Clinch River microeukaryotes for an adjacent study suggested a large proportion of the microbiome was fungal (unpublished data) which led us to hypothesize that mussels could be using them as a food source. There are few published accounts of mussels consuming fungi. The earliest is a study where, *C. iris* juveniles bred in captivity were exclusively fed “commercial yeast” (presumably *Saccharomyces cerevisiae*) and died within 8 days of the beginning of the feeding regimen^51^, suggesting they derived no nutrition from the yeast and/or may have been poisoned by them. In contrast, *Elliptio complanata* were found to clear spores of 7 common river hyphomycetes from four families (Leotiaceae, Discinellaceae, Nectriaceae, and Halosphaeriaceae) at clearance rates comparable to algal consumption^54^. They also found that the rate of removal differed by fungal species, suggesting selective feeding.

Both of our experiments demonstrated that unionids can clear fungi from the plankton, providing more evidence that mussels can consume and presumably derive nutrition from fungi. Four of the mussel species we tested, appear to have cleared fungi more quickly than a fifth species suggesting there could be selective feeding. Other evidence of possible selective feeding came from taxonomic analyses of the planktonic fungi. The fungal families Mycosphaerellaceae and Zygophlyctidaceae were cleared from river water by all five mussels within 24 h but not Didymosphaeriaceae, Didymellaceae or Cladosporiaceae. It is tempting to hypothesize that these differences could be due to spore size but Bälocher and Brendelberger^53^ found that there was no correlation between fungal spore size and its rate of consumption by a freshwater mussel. There is also the possibility that consumption could have been due to the fungal life stage, i.e. whether they were present as spores or hyphae. This possibility can only be addressed with future experiments.

The family Mycosphaerellaceae are plant/agricultural pathogens^55^ and Zygophlyctidaceae is in the phylum Chytridiomycota which are common in aquatic systems and contain pathogens such as *Batrachochytrium dendrobatidis* which is responsible for herpetological die-offs^56^. The fact that mussels can clear these fungal spores from the phytoplankton provides another argument for the re-establishment of mussel beds and their critical contribution to the ecological health of aquatic systems.

Metabarcoding of the fungi in the retention pond did not clearly demonstrate that mussels were clearing them from the plankton which contradicted the qPCR results from the same set of feeding experiments. This suggests that qPCR and metabarcoding could be detecting different barcodes or that the metabarcoding process inadvertently overemphasized taxonomically identifiable fungi, of which the majority within this body of water was Didymosphaeriaceae, which was not a preferred food source. These contradicting results suggest further experimentation is warranted.

### Bacterial consumption

In our first experiment, the mussels we tested did not clear a significant amount of bacteria from river water within a 24 h incubation period. This led us to hypothesize that the mussels may only ingest bacteria after their preferred food sources have been depleted, so in a second experiment the mussel incubation time was extended to 4 days. The amount of detectable bacteria dropped only in the presence of *O. pectorosa*, but not the other 4 mussels. These results are intrigueing and offer the possibility that some mussel species can consume bacteria but others either do not consume bacteria or do so less efficiently. For this mussel species, in particular, further experiments investigating our results may be warranted. Following a mussel mass mortality event in the Clinch River, TN that began in summer of 2016, *O. pectorosa* was documented to be more severely impacted than other congener species and causes are still being investigated to explain this phenomenon^9,57^. Although links to pathogens and parasites have some empirical support, dietary preferences and energy needs have not yet been fully investigated. It is possible that some food preference with regard to bacteria could offer more insight.

The consumption of bacteria by mussels is debatable. Early supposition suggested and several isotope biomarker studies offered evidence that mussels ingest bacteria as a primary source of carbon^21,25,30^. These results have been questioned, however, as controlled feeding experiments by Gatenby et al.^51^ demonstrated that bacteria did not contribute to the growth of *C. iris* and others have pointed out that bacteria lack required nutrients such as long-chain fatty acids, sterols, and amino acids important to sustain mussel growth^21^. In addition, studies offering evidence of bacterial consumption have been based on isotope analyses which cannot distinguish sources of biomolecules so it is possible that mussels could have indirectly consumed bacteria by eating Alveolate bacterivores who had recently had a bacterial meal^29^. Other studies have relied upon the presence of bacterial fatty acids such as C18:1 to assess the consumption of bacteria but these fatty acids are also abundant in algal chloroplasts^58^ raising the possibility that algal fatty acids could have been mistaken as bacterial. Freshwater mussels also sustain an endosymbiotic gut microbiome^59^ which is established from bacteria filtered from the environment that are not digested^60,61^. Therefore it is possible that some earlier studies mistook the microbiome for consumed bacteria^23^. These varied results have led some to propose that bacterial consumption is linked to life stage where only adult mussels rely on bacteria as a carbon source^21,25,30^ whereas juvenile mussels mainly consume microalgae. Others posit that bacterial consumption by mussels depends upon their environment with those residing in small temperate streams relying more heavily on the consumption of bacteria than those in large productive rivers^21,23,25,62^. In our experiments it appears that at least one mussel, *O. pectorosa*, may consume planktonic bacteria after it has depleted preferred algal and fungal meals. The length of our feeding experiment was limited to 4 days out of concern for the health of the mussels but it is possible that other species might consume bacteria in response to prolonged starvation.

### Selective feeding

Evidence presented in this paper suggests that freshwater mussels can preferentially feed on Stramenopiles, some families of fungi over others, and both over bacteria. This is consistent with previous studies where freshwater unionids were observed to preferentially feed on algae over bacteria^19,21,30,50^ or organic living materials over inorganic debris within the feeding substrate^23^.

Studies of marine species, such as *Mytilus edulis*, and zebra mussels have found evidence of diet selectivity^15–18,63^. Freshwater mussels can also differentiate between particle sizes as well as nutritional phytoplankton over non-nutritional inorganic particles^50^. Therefore it is possible that particle size can play a role in selective feeding but this is most likely not the only factor. For example, Bärlocher and Brendelberger^54^ found that spores of different fungal species were cleared at different rates but there was no correlation between the fungal spore size and its rate of removal. Another factor in feeding was demonstrated by Mistry and Ackerman^50^ who reported that four species of mussels (*L. siliquidea*, *L. fasciola*, *Ligumia nasuta*, and *C. iris*) differentially fed on algae depending upon algal cell concentration and water velocity.

#### Removal of ions

We hypothesized that mussels would not have a measurable effect upon water chemistry. In our experiments, however, we found that water ion content, measured as a function of conductivity, was significantly reduced by the presence of mussels in both experiments. Mussels are known to be affected by heavy metal contamination^64^ and can remove nitrite, nitrate, and ammonia^32^ presumably incorporating them into their shells^65^. The ion content of waters collected from the Clinch River and AWCC pond were not assayed in this study but could form the basis of a future project.

## Summary and conclusions

In this study we describe the first assessment of the microbial planktonic diet of freshwater mussels using environmental DNA analysis. We tested two hypotheses 1—Siphon feeding Unionid mussels can select preferred microbes from mixed plankton, 2—Different mussel species exhibit dietary niche differentiation, and failed to reject them. Additionally, these experiments revealed that *Ortmanniana pectorosa* consumed a wider array of bacteria and fungi than other species we tested. This is significant as it demonstrates a unique dietary niche for this species within multi-species aggregates and also because it provides another factor when considering why *O. pectorosa* is distinctly affected in mass mortality events in the Clinch River.

The characterization of mussel diet using this approach is novel in that it does not require the sacrifice of potentially imperiled mussel species and can identify the specific microbial taxa that are consumed. Among the limitations, however, is the assumption that the disappearance of microbes from the water means the mussels consumed those microbes. Control tanks containing no mussels were included in our experimental design to determine if microbes were being removed by the mussels rather than succumbing to the experimental conditions but we can only surmise that the mussels derived nutrition from the microbes as they cleared them from the plankton. Another drawback is that only suspension feeding of planktonic microbes was tested. The importance of pedal feeding of solid substrate has been demonstrated by multiple studies^23,26,66^ some of which provide evidence that in some environments mussels derive more nutrition from microbes inhabiting the benthos than from plankton. Feeding habits and selectivity of benthic organisms is an important question that could be addressed in future metabarcoding experiments. Finally, the taxonomic identifications in all metabarcoding studies are limited by the databases and scripts used in the identification process.

As freshwater mussels are considered one of the most imperiled group of organisms, our results can aid in conservation efforts, as knowing the preferred diet composition of freshwater mussels can be used to manage mussel health. Anthropogenic habitat degradation continues to jeopardize the mussel fauna of the Clinch River, VA and significantly impedes species recovery^67^. The conservation status of each species studied in this project are as follows: *Cambarunio iris* (currently stable); *Ortmanniana ligamentina* (currently stable); *Ortmanniana pectorosa* (species of concern); *Lampsilis fasciola* (currently stable); *Lampsilis ovata* (species of concern)^1^. Therefore, it is crucial to the success of conservation efforts to understand the diet composition of freshwater mussels.

### Supplementary Information

Supplementary Figure S1.

Supplementary Figure S2.

Supplementary Legends.

Supplementary Table S1.

Supplementary Table S2.

Supplementary Table S3.

Supplementary Table S4.

Supplementary Table S5.

Supplementary Table S6.

Supplementary Table S7.

Supplementary Table S8.

## Data Availability

Metabarcode raw sequence data are archived at NCBI’s SRA database (PRJNA1040762) and the University of Virginia Library’s Dataverse repository 10.18130/V3/RJ4EWF.
