# Supplementary figures and images for: Freshwater mussels prefer a diet of stramenopiles and fungi over bacteria
Source: Sci Rep. 2024 May 25;14:11958. doi: 10.1038/s41598-024-62245-2 (PMC11127930; doi:10.1038/s41598-024-62245-2)

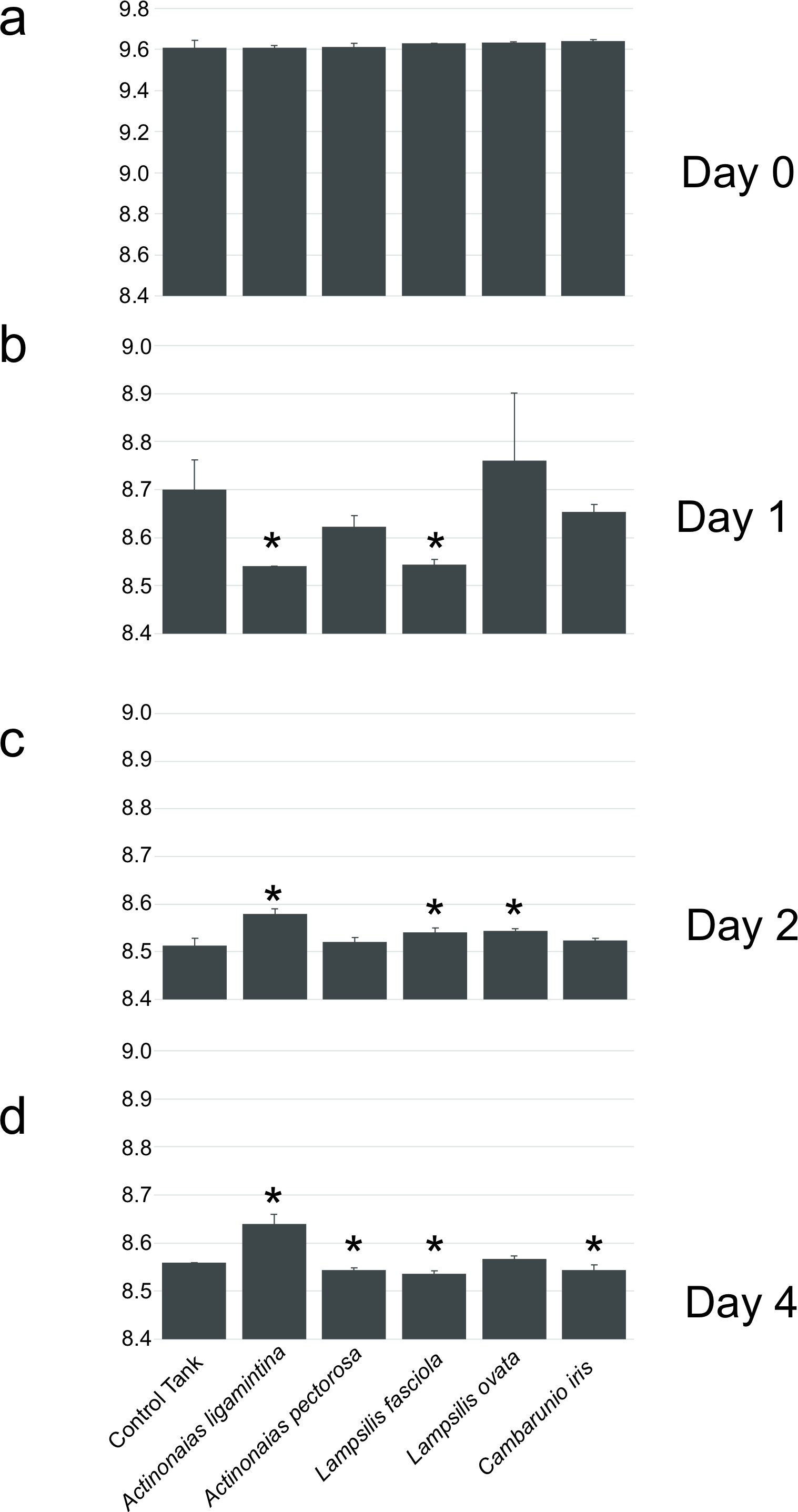

Supplement: Supplementary file 1 — Supplementary Figure S1. [file 41598_2024_62245_MOESM1_ESM.jpg]

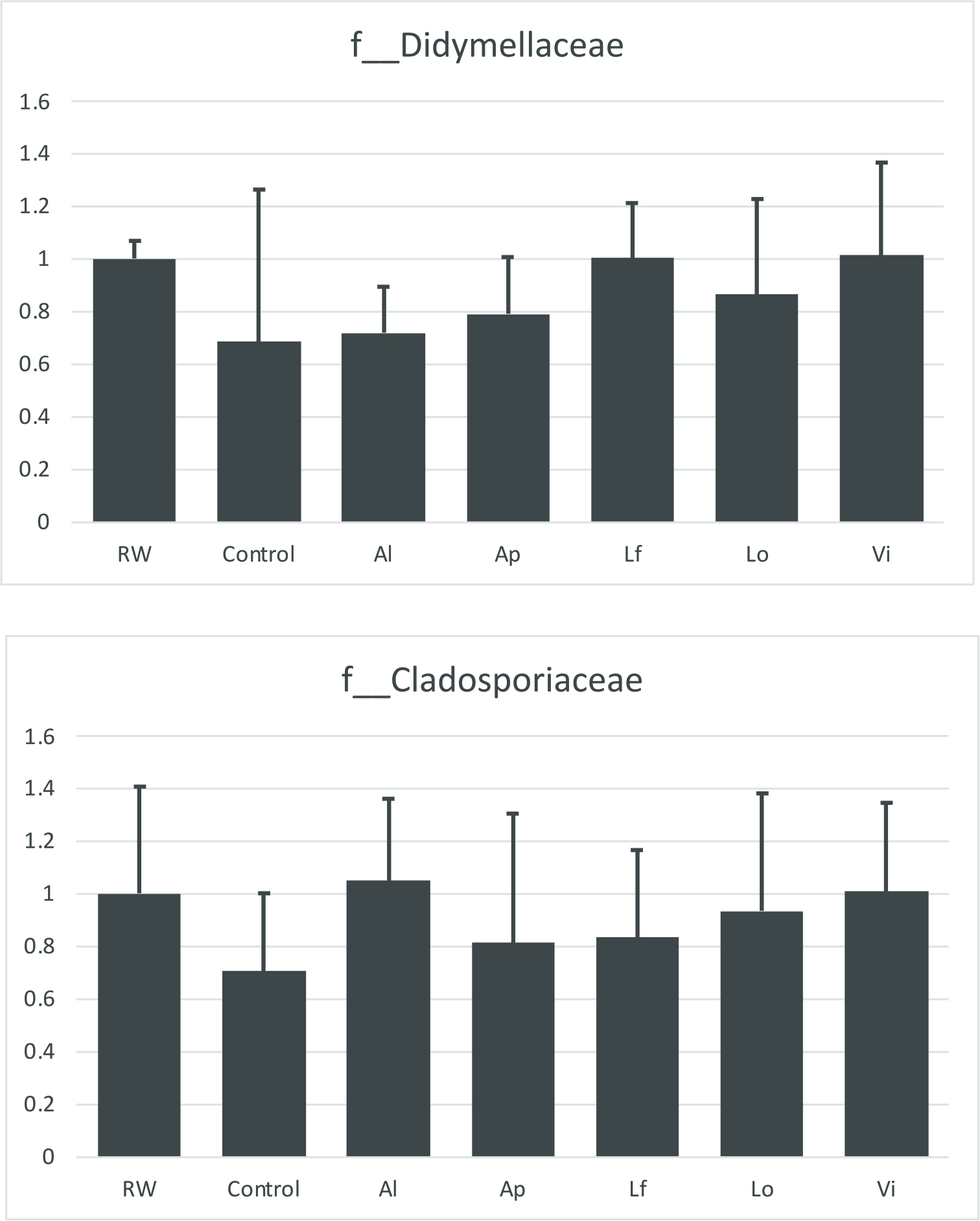

Supplement: Supplementary file 2 — Supplementary Figure S2. [file 41598_2024_62245_MOESM2_ESM.jpg]
