# Supplementary material for: Freshwater mussels prefer a diet of stramenopiles and fungi over bacteria
Source: Sci Rep. 2024 May 25;14:11958. doi: 10.1038/s41598-024-62245-2 (PMC11127930; doi:10.1038/s41598-024-62245-2)
Supplement: Supplementary file 3 — Supplementary Legends. [file 41598_2024_62245_MOESM3_ESM.docx]

**Supplementary Information**

Supplementary Figure S1. Fungal Families That Were not Cleared by Mussels.

Supplementary Figure S2. pH of AWCC Pond Before and After Incubation with Mussels.

pH was measured immediately after water was placed into the tanks (Day 0) and then 1, 2, and 4 days after mussels were introduced into the tanks. Error bars represent standard deviation. Asterisks represent p-values equal or lower than 0.05 compared to river water (one-way ANOVA). Plus symbols represent p-values equal or lower than 0.05 compared to river water (one-way Kruskal-Wallis).

Supplementary Table S1. Experiment 1 Statistics

Supplementary Table S2. Sizes of Mussels Used in Both Experiments.

Supplementary Table S3. Experiment 2 Statistics

Supplementary Table S4. Bacterial Phyla Present in the Clinch River Phytoplankton.

Supplementary Table S5. Microeukaryotic Phyla Present in the Clinch River Phytoplankton.

Supplementary Table S6. Fungal Phyla Present in the Clinch River Phytoplankton.

Supplementary Table S7. Bacterial Phyla Present in the AWCC Retention Pond Phytoplankton.

Supplementary Table S8. Fungal Taxa Present in the AWCC Retention Pond Phytoplankton.
